# Content-Based Medical Image Retrieval and Intelligent Interactive Visual Browser for Medical Education, Research and Care

**DOI:** 10.3390/diagnostics11081470

**Published:** 2021-08-13

**Authors:** Camilo G. Sotomayor, Marcelo Mendoza, Víctor Castañeda, Humberto Farías, Gabriel Molina, Gonzalo Pereira, Steffen Härtel, Mauricio Solar, Mauricio Araya

**Affiliations:** 1 Radiology Department, Clinical Hospital University of Chile, University of Chile, Santiago 8380453, Chile; camilosotomayor@ug.uchile.cl (C.G.S.); gpr.pereira@gmail.com (G.P.); 2 Center for Medical Informatics and Telemedicine, Institute of Biomedical Sciences, Faculty of Medicine, University of Chile, Santiago 8380453, Chile; vcastane@uchile.cl (V.C.); shartel@uchile.cl (S.H.); 3 Department of Electronic Engineering, Federico Santa Maria Technical University, Valparaíso 2340000, Chile; 4 Department of Informatics, Federico Santa Maria Technical University, Santiago 8380453, Chile; marcelo.mendoza@usm.cl (M.M.); humberto.farias@usm.cl (H.F.); gabriel.molina.12@sansano.usm.cl (G.M.); mauricio.solar@usm.cl (M.S.); 5 Department of Medical Technology, Faculty of Medicine, University of Chile, Santiago 8380453, Chile

**Keywords:** clinical, content-based image retrieval, education, imaging, interactive visual browser, query-by-example, research

## Abstract

Medical imaging is essential nowadays throughout medical education, research, and care. Accordingly, international efforts have been made to set large-scale image repositories for these purposes. Yet, to date, browsing of large-scale medical image repositories has been troublesome, time-consuming, and generally limited by text search engines. A paradigm shift, by means of a query-by-example search engine, would alleviate these constraints and beneficially impact several practical demands throughout the medical field. The current project aims to address this gap in medical imaging consumption by developing a content-based image retrieval (CBIR) system, which combines two image processing architectures based on deep learning. Furthermore, a first-of-its-kind intelligent visual browser was designed that interactively displays a set of imaging examinations with similar visual content on a similarity map, making it possible to search for and efficiently navigate through a large-scale medical imaging repository, even if it has been set with incomplete and curated metadata. Users may, likewise, provide text keywords, in which case the system performs a content- and metadata-based search. The system was fashioned with an anonymizer service and designed to be fully interoperable according to international standards, to stimulate its integration within electronic healthcare systems and its adoption for medical education, research and care. Professionals of the healthcare sector, by means of a self-administered questionnaire, underscored that this CBIR system and intelligent interactive visual browser would be highly useful for these purposes. Further studies are warranted to complete a comprehensive assessment of the performance of the system through case description and protocolized evaluations by medical imaging specialists.

## 1. Introduction

Nowadays, imaging plays a central role in medicine. Large amounts of imaging data are constantly generated in daily clinical practice, leading to continuously expanding archives, and ever progressive efforts are being made across the world to build large-scale medical imaging repositories [1,2]. This trend is in line with the increasing medical image consumption needs, which have been studied and categorized into four groups: patient care-related, research-related, education-related, and other [3].

In the era of big data, however, navigating through large-scale medical imaging archives is becoming, correspondingly, increasingly troublesome. Browsing any available, large-scale medical imaging repository through a conventional text-based search engine is time-consuming, severely hampered if the repository lacks curated or expert-annotated metadata, the search results display options are limited. Conversely, the need for collecting curated or expert-annotated metadata may, in turn, be preventing the building of large, multi-center, international medical imaging repositories that meet the medical imaging needs of today. In this scenario, there is an enormous need for efficiently archiving, organizing, managing, and mining massive medical image datasets on the basis of their visual content (e.g., shape, morphology, structure), and it may be expected that this demand will only become more substantial in the foreseeable future.

Accordingly, attempts have been made over the last few decades to complement search strategies of conventional text-based engines by means of advancing image content-based repository indexation technology as this may lead to novel search engine possibilities [4]. Several approaches have been used to develop content-based image retrieval (CBIR) systems that allow for automatic navigation through large-scale medical image repositories [4]. Such promising capability fuels research efforts in the fields of computer vision and deep learning. 

Formally, a CBIR system is a quadruple {*D, Q, F, R (q_i_, d_j_*)}, where: (i) *D* is a set composed of representations for the images in a given collection, (ii) *Q* is a set of representations for user information needs, operationally known as queries, (iii) *F* is a representational framework that allows images, queries, and their relationships to be jointly modeled, and, finally, (iv) *R(q_i_, d_j_)* is a ranking function which associates a real number with a query *q_i_* in *Q* and an image *d_j_* in *D*. The ranking defines an ordering among the images in a given collection regarding the query *q*.

Regarding *F*, this framework can be learned using supervised or unsupervised approaches. In supervised approaches, the representational framework has a grounded dataset made up of pairs of images and queries along with their respective ranking scores. The main limitation of these approaches is the difficulty of building a large volume of curated data that allows the system to generalize for new queries and images not considered during the training phase of the system. On the other hand, unsupervised approaches do not require a grounded dataset to train the representational framework. Instead, this type of system is typically based on distances between vector representations of images and queries. We take this second approach for this proposal.

CBIR systems have the potential to deliver relevant technology for clinical imaging through several use cases in medical education, research, and care, including clinical diagnostics [5]. In radiology, for instance, it has long been shown that CBIR can facilitate diagnosis, especially for less experienced users such as radiology residents, but also for radiologists in cases of less frequent or rare diseases [6]. Noteworthy, relatively less explored has been the possibility that image content-based repository indexation technology brings about to augment search results display options. This indexation approach empowers the development and application of intelligent similarity maps to display search results, which may further boost efficiency of navigation through large-scale medical imaging repositories. If integrated with a PACS (Picture Archiving and Communication System), this approach would make it possible to perform novel, automatic, independent, medical inter-consultations using medical image content with similar cases available in the local and other linked archives without requiring the user to enter a priori keywords to drive the search towards the best visual content match. Furthermore, such a CBIR system and intelligent and interactive visual browser, thoughtfully designed to be fully interoperable with healthcare systems according to international standards, may stimulate a burst of novel opportunities for medical education, research, and care. 

The literature on this topic has identified use cases even when technical limitations would only allow CBIR based on shallow features such as shape and texture, and at a time when medical imaging fields have counted on a narrow set of digital images of limited resolution. Novel deep learning architectures have paved the pathway towards unleashing the potential for innovative applications to solve long-standing medical image search problems. Moreover, features from deep convolutional neural networks have yielded state-of-the-art performance in CBIR systems by efficiently overcoming long-standing computational shortcomings [7,8,9]. With current computing capacity increasingly supplying powerful image analysis techniques, a new surge of capabilities and applications have been devised. Novel deep learning architectures have also empowered internal image representation learning, specifically, latent representations, which can be used to implement ground-breaking image content search engines [10,11,12]. 

Looking towards their implementation in the daily clinical workflow, however, there remain technical challenges. Large-scale repositories, reciprocally, are needed for CBIR systems to deliver appealing search results. For that purpose, in turn, local teams with experience in the use of healthcare integration standards are required. For multi-center collaborative efforts, there is also needed a data extractor inside each institution to anonymize, and transfer and convert data to a standardized semantic term. 

The aim of the present project was to develop a CBIR system using learned latent image representation indexation, with a visual content, similarity-based, intelligent and interactive visual browser for efficient navigation. The system was developed using international standards to be fully interoperable to ease integration into routine clinical workflow and, thus, support current medical image demands throughout education, research and clinical care. 

## 2. Materials and Methods

### 2.1. Building an Interoperable, Standardized and Anonymized Medical Image Repository 

Integration with the hospital (Clinical Hospital University of Chile, Santiago, Chile) to have continuous feeding of medical images and metadata was composed of different microservices. First, a Mirth Connect integration system was used to receive data in different health standard types, DICOM channels, HL7 messages, and HL7-FHIR messages. Mirth Connect was integrated to an Anonymizer service, which eliminates all patients’ personal data and extracts healthcare data to feed an FHIR server using international standardized health terms (SNOMED-CT, ICD-9, ICD-10). This allows the development of an interoperable and standardized repository. The Institutional Review Board approved (METc 2020/035, on 8 July 2020) the storage and management of thorax computed tomography in this repository. All procedures were conducted in adherence to the declarations of Helsinki.

### 2.2. Deep Learning-Based Medical Image Indexation and Retrieval Strategy

Two image processing architectures based on deep learning were combined. The first, CE-Net (Context Encoder Network) [13], was used to build a representation of the input image in a lower dimensional space, i.e., a latent representation. Using these image embeddings, a second neural architecture was trained, Xception [14], which is capable of learning a new representation of the input image. This architecture is trained to solve a diagnosis classification task, which helps group images that coincide with their initial diagnosis. 

#### 2.2.1. CE-Net

The image segmentation architecture CE-Net [13] was used first, which allows 2D medical images to be processed. The CE-Net architecture is an extension of the U-Net architecture [15], which is an encoder-decoder architecture. Encoder-decoder architectures work with a tandem of neural layers, wherein the first architecture block, the encoder, is a sequence of layers responsible for building a representation of the input image in a lower dimensional space, known as latent space. The second architecture block, the decoder, is another sequence of layers responsible for transforming the encoding from latent space to original space, recovering the original dimensionality of the image. The layers of the U-Net architecture are convolutional, introduce max-pooling operators, and add residual connections to prevent the network from the loss of information between layers. This allows the construction of lower dimensional representations that retain the most important information from the encodings obtained in the previous layers. The architecture parameters are adjusted in such a way as to minimize the reconstruction error (defined as the difference between the original image and the reconstructed image). Thus, the encoder-decoder architectures are intended to encode the images in the latent space. The CE-Net architecture extends the U-Net architecture by incorporating two processing modules: the dense à-trous convolution (DAC) and the residual multi-kernel pooling (RMP) module. Both modules were designed to capture high-level characteristics, preserving spatial information throughout the encoder-decoder architecture. These representations were used to feed the second neural architecture used by the system, the Xception. 

#### 2.2.2. Xception

Xception [14] is an architecture that extends the Inception [16] architecture used in image classification. Classification models based on deep learning achieve good performance by incorporating representation learning mechanisms that enable adapting of these representations to the tasks for which they are trained. The Inception architecture uses modules based on convolutional operators that capture short-range dependencies in the input image; this allows learning of a new representation of the input image by identifying patterns between the pixels of the original image, which is useful for a better representation. The Xception architecture extends the Inception architecture by incorporating convolutional layers that allow capturing long-range dependencies, which has allowed this architecture to obtain good results in classification problems.

Yet, one of the disadvantages of architectures such as Xception, is that they have many parameters and, therefore, require a large volume of data to be trained. The Xception architecture was validated on an internal Google dataset, called JFT, which has more than 350 million high resolution images with labels on the order of 17,000 classes. The availability of labeled imaging data at this volume is unlikely to be reached in the medical field in the near future. Because the scale of the repositories with which this project works is much smaller, training an Xception architecture for medical images from scratch is not feasible. 

Therefore, a different approach was introduced that allows reducing of the gap between the need for large volumes of data to use deep learning, and the actual availability of medium-scale datasets in the clinical setting. Two architectures trained in different tasks were combined. The CE-Net architecture was used to segment the images of the repository, and the latent representations were used as pre-trained vectors to adjust the Xception architecture according to diagnosis. By segmenting the images and working with their latent representations, their variability was reduced and placed in a common representation space, which provides better generalization abilities to the Xception because, instead of working with the original images, it works with images with reduced dimensionality. Thus, the Xception network can work with fewer parameters when solving the diagnostic classification task, making it possible to avoid the risk of overfitting attributable to limited volumes of data. Another advantage of this way of proceeding is that it enables working on images of different types. Since the CE-Net architecture builds a common representation space for images of different types, the Xception network can process these images indistinctly, solving the diagnostic classification problem on heterogeneous image sources.

Note that Xception is a supervised method. To solve the classification problem according to diagnosis, the model must encode the images to consistently activate the feature maps of the output layer in correspondence with the diagnosis. Accordingly, the encodings of the images that coincide in diagnosis decrease their relative distance, producing a clustering effect in the embedding space. Our representational framework takes advantage of these Xception features to improve the system’s search abilities in the target classes.

#### 2.2.3. Ce-Net + Xception Assembling

We tested latent embeddings extracted from the DAC and RMP blocks of the Ce-Net, the latter delivering the best results in our system. The encoding extracted from the RMP block was fed into the Xception network. Accordingly, instead of working directly on the images, the Xception network works on the encodings of the images delivered by the Ce-Net RMP block. The RMP block gathers information from the DAC block using four different-sized pooling kernels. These features are fed into a 1 × 1 convolution to reduce the dimension of feature maps. Finally, these features are concatenated with the original features extracted from the DAC block, producing an encoding. 

The Ce-Net processes images of 512 × 512 dimensions. The RMP block produces encodings with 384 × 344 dimensions. This encoding is ingested into the Xception, which outputs an activation map of the same dimensions. Both encodings, of the RMP block and for the output activation of the Xception, are flattened and concatenated, producing a one-dimensional embedding with 264,192 entries.

According to the formal definition of a CBIR system provided in Section 1, each quadruple element corresponds to the following elements of our framework. *D* is the set of images indexed by our system, *Q* is an unbounded set of images used to query our system, *F* is the representational framework defined by the Ce-Net + Xception ensemble, and *R(q_i_, d_i_)* is the distance computed by the nearest neighbor query engine. Note that the queries are unseen images, i.e., medical images not used during the representation learning training phase. In order to create the query encodings, we need to feed these images into the representational framework. Accordingly, for each query image, their embeddings are retrieved from the RMP block and the last feature activation map of the Xception network. Finally, these embeddings are used as query vector representations to feed the query engine.

One of the characteristics of our ensemble is that it combines two different capabilities in a single representational framework. On the one hand, the Ce-Net encodes images using convolutional filters of different sizes, which allows it to represent tumoral lesions of different sizes. This characteristic is mainly due to pooling kernels, which are used in the DAC and RMP blocks of the architecture. On the other hand, the Xception network allows images to be grouped according to diagnosis. The effect that this network produces in the latent space is to cluster the encodings, reducing the relative distances between the images that coincide in diagnosis. This capacity provides the system with better detection capabilities in the target classes. Due to the above, it is expected that images with lesions of different sizes or types of tissues, but that coincide in diagnosis, will cluster in the embedding space.

#### 2.2.4. Training

The CE-Net can be trained for medical image segmentation tasks by showing original segmented image pairs to the network at the input and output of the network. This requires having a set of medical images together with their segmentation masks, generated by medical imaging specialists. To do this, we trained CE-Net with public datasets of different types of medical images. 

The SARS-CoV-2 CT scan dataset was used to train the Xception network (SARS-CoV-2 CT-scan dataset: A large dataset of real patients CT scans for SARS-CoV-2 identification. Available online: https://www.medrxiv.org/content/10.1101/2020.04.24.20078584v3, accessed on 27 July 2021). The dataset contains 1252 CT slices that are positive for SARS-CoV-2 infection (COVID-19) and 1230 CT slices for patients non-infected by SARS-CoV-2. Since each scan contains multiple slices, and some do not show ground-glass or other evidence of COVID-19, we used the Ce-Net to sample CT slices. The segmenter allowed CT slices to be sampled for COVID-19 patients who had evidence of ground-glass or pleural effusion. One axial slice was sampled per scan, maintaining the balance between healthy and sick patient slices.

As a validation set, the COVID-CT-dataset (COVID-CT. Available online: https://github.com/UCSD-AI4H/COVID-CT, accessed on 27 July 2021) was used as a testing set. The dataset contains 349 CT slices from 216 COVID-19 patients and 463 non-COVID CT slices. The dataset contains images acquired with different media, for example, CT slices post-processed by cell phone cameras and some images with very low resolution. For these reasons, the dataset represents the real conditions of image acquisition for a system of this kind.

To generate a balanced set of queries, the Clinical Hospital of the University of Chile supported us with eight CT scans where half of them had suffered from COVID-19. From these CTs, 25 slices with COVID-19 and 25 slices without COVID-19 were extracted. Each of these queries was used to query our system.

As the Ce-Net is a network with many parameters, and to avoid overfitting, we initialized their weights using ImageNet samples [17] (ImageNet. Available online: https://image-net.org/, accessed on 27 July 2021).

#### 2.2.5. Validation 

The performance of our search system was evaluated using precision and recall measures. To compute these metrics, the CT slices’ ground labels are considered according to SARS-CoV-2 diagnosis, counting matches between image examples labels and their list results. This proposal was evaluated considering four alternative methods:

- Ce-Net [13]: Corresponds to a search system based on the encoding of the testing images obtained from the Ce-Net using the RMP block.
- Xception [14]: Corresponds to a search system based on the encoding of the testing images obtained from the Xception using its last layer.
- U-Net-ME (manifold embeddings): Corresponds to a search system based on the encoding of the testing images obtained from the architecture of Baur et al. [18], which extended U-Net and was trained for 5 epochs for segmentation and then 5 epochs with the manifold embedding loss. The embeddings were obtained using the last layer of the architecture.
- U-Net-ML (mixed learning): Corresponds to a model based on [18], for searches over the encoding of the testing images obtained from a U-Net architecture. Trained for 5 epochs for segmentation, then 5 more epochs for classification. We conducted tests with several layers of the encoding but those that obtained the best results were the embeddings obtained using the last layer of the architecture.

**Figure 1:**
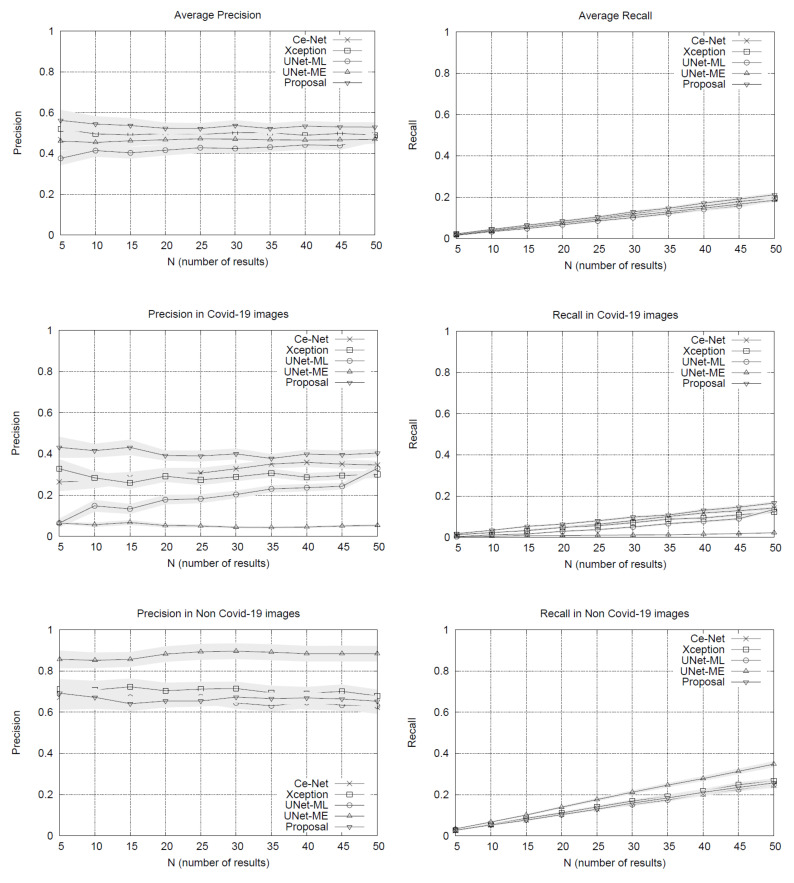
Precision and recall scores of the methods evaluated in this study. The plots at the top show the whole set of images’ performance, while the plots at the bottom show the results disaggregated by class.

The results of the experiments are shown in Figure 1. The performance plots on the whole set of testing queries (at the top of Figure 1) show that our proposal outperforms the other methods in precision. As we might expect, the precision drops slightly as the list of results grows. The variance around the mean precision also decreases gradually. The recall of all the methods is quite similar, reaching around 20% in lists with 50 image results.

By separating the testing set between COVID-19 and non COVID-19 queries, the results in Figure 1 show that our method obtains advantages over other methods when using queries of patients with COVID-19, surpassing by a significant margin the most direct competitor, the Xception network. The other methods have lower performances. U-Net-ME performs well in the healthy patient class. However, this model exhibits overfitting to this class as its performance in the COVID-19 class is very low. Our proposal surpasses the other methods in COVID-19 images regarding recall rates, while U-Net-ME generates a better recall in images of healthy patients. The results confirm that our proposal is suitable for searching for images of COVID-19 patients, surpassing all its competitors in precision and without generating overfitting to any of the classes.

With the Ce-Net + Xception network validated, latent vectors were indexed in a nearest neighbors query engine, which is the basis of the intelligent visual browser.

### 2.3. Intelligent Interactive Visual Browser for Medical Images

The data ingestion pipeline (content + metadata) to the PACS has been systematically tested in its different stages, with different image sources. The software architecture design of the CBIR system was developed following a model of microservices grouped in the back-end and front-end of the system. These services are communicated via REST API (representational state transfer) in order to have a scalable system for a correct incorporation of new data and their respective descriptive metadata. This pipeline makes available the metadata information necessary for the results of the CBIR system. In the case of content (images), the procedures for their incorporation into the PACS go together with the metadata in the data ingestion pipeline, thus being available as potential search results for the CBIR system. The design of the system allows us to function in a decoupled way to the nature of the image for which the architecture of neural networks are designed to work. In the event that the image format is changed, it would only be necessary to update the network architecture.

#### 2.3.1. Back-End

The combination CE-Net + Xception constitutes the back-end of the browser. The back-end provides a vector representation of all the images in the repository, i.e., the latent representations constructed using the CE-Net + Xception allow obtaining continuous and dense vectors of the same dimensionality for all the images in the repository. It is noteworthy that combining these architectures makes metadata availability requirements more flexible since none of the networks require metadata for ingested imaging datasets. This means that images of different types could be received without metadata, and all of them would have a latent representation in the same latent space that Xception uses for classification. Moreover, images of latent representations retrieved by Xception are expected to be separated by types of images (as a result of the CE-Net segmentation model) and by diagnosis (as a result of the Xception classification model). The clustering hypothesis according to type of image and diagnosis is supported by the combination of both architectures in tandem. This means that while the segmenter allows different types of medical images to be represented in the same representation space, the Xception network helps to separate them by diagnosis. It is worth mentioning that if diagnostic metadata is not available, the Xception network will obtain the representation of the unlabeled images using them as a testing partition.

#### 2.3.2. Front-End

The front-end is responsible for enabling the search engine on the repository. To do this efficiently, the images are indexed using an efficient data structure that allows searching for close neighbors. This data structure is Multiple Random Projection Trees (MRPT) [19], which is considered to be state-of-the-art in the approximate search for close neighbors. MRPT allows the building of a search index with recall guarantees. This means that we can indicate a minimum recall rate and the structure is built in a way that satisfies this restriction. This element is important because if the repository grows in volume, an index that scales to a larger volume of data will be required. Once the index has been built, queries of close neighbors can be run. The user determines the number of nearest neighbors from the repository and returns the identifiers of the corresponding images. Queries are new images that do not need to be labeled, i.e., they do not need to be entered with any accompanying text. To enter it as a query, the image is first segmented using the CE-Net network pre-trained on the repository images. After retrieving its latent vector and ingesting it in the Xception network, the latent vector is retrieved from the Xception network, which is used as a representation of the image. This vector is in the same latent space as the images indexed in MRPT, so it can be used as a query vector to retrieve its nearest neighbors. As the representation is built on the same pipeline with which the repository images have been processed, the nearest neighbors will correspond to images that are similar in content, both in segmentation structure and diagnosis.

**Figure 2:**
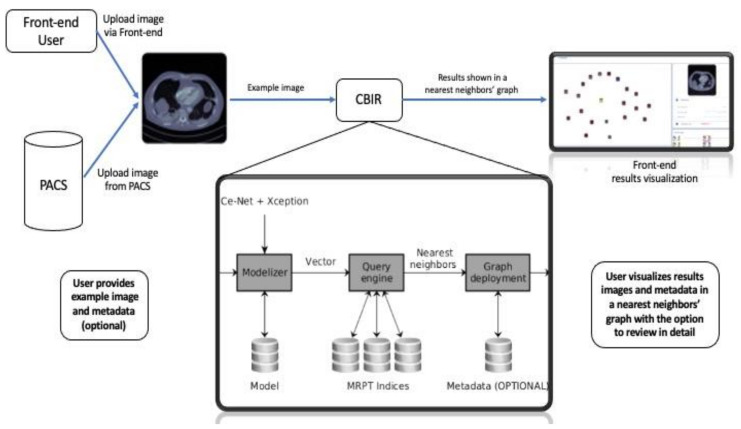
Pipeline of the proposed system. A query image is provided via the front-end or sent directly from the institution’s PACS. The system has a query pipeline on the front-end of the intelligent interactive visual browser for medical images. A new image is processed using the CE-Net + Xception architectures to obtain its vectorized latent representation. The query engine accesses the repository indexes, retrieving the nearest neighbors images from the repository, which are displayed by the interactive visual browser for medical images (as shown further in Figure 3). Metadata, whenever available, is also displayed. Then, the nearest neighbors’ graph is shown in the front-end to visualize the results.

Figure 2 shows the pipeline of our proposed assistance system, including the query pipeline implemented on the front-end to enable the intelligent interactive visual browser for medical images. 

Since the browser is based on proximity searches, creating an ego network around the query image would provide valuable information to the user as to understand the different groups of images that make up the set of results. Because displaying the results according to similarities may be expected to be more informative than conventional displaying fashion in the form of a list, the visual browser was designed taking into consideration that once the nearest neighbors of the query image are retrieved, the results could be displayed by implementing a proximity graph.

### 2.4. Results Display

**Figure 3:**
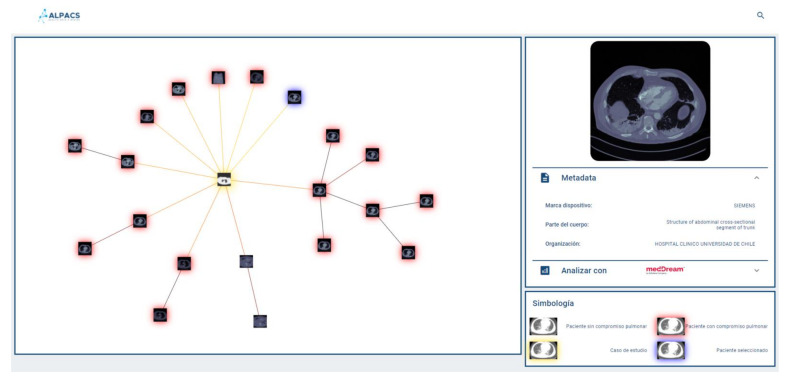
A new image is used to formulate a query. The user has the option to also include a diagnosis or other keywords. The query engine displays nearest neighbors’ graph (with and without pulmonary findings shown with red halo and without colored halo, respectively) centered around the query image (yellow). The user can select an image from the graph, which displays the image’s metadata tab, if the metadata is available. The system is currently available in Spanish (*marca dispositivo*, device brand; *parte del cuerpo*, body part; *organización*, organization; *simbología*, symbology).

A key aspect of our visual browser concerns its fashion for displaying search results. A graphic library called VivaGraph (VivaGraph. Available online https://github.com/anvaka/VivaGraphJS, accessed on 27 July 2021) was selected, in JavaScript, which allows rendering results’ graphs in near real-time. The VivaGraph library allows a graph display with thousands of nodes in fractions of seconds, making it ideal for the purposes of this search engine. The rendering of the graph consists of generating a node for each result, and joining the results with edges whose length is inversely proportional to their proximity. In this way, the closest results are grouped into clusters and the most distant are more distantly displayed. The rendering is defined by a layout algorithm. A Force Atlas layout is used that produces more compact graphs than other layouts, which is useful for the tool. The graph is responsive, i.e., it allows the user to select an image and display a metadata box that complements its description (if available). Whenever metadata is available, the viewer will show it in a responsive selection box, which delivers a tab of an image selected by the user on the proximity graph. The system allows the user to provide text (e.g., diagnosis of the query image) as part of the repository consultation, in which case it will be used to filter out the results returned by the query engine. This case is called hybrid search (content + metadata). If the user does not provide text, the query is performed by using a content-only search (no metadata). Figure 3 shows a query graph displayed using our system.

### 2.5. Survey among Professionals of the Healthcare Sector

To explore the potential applications that professionals of the healthcare care sector would foresee for a CBIR system with an intelligent interactive visual browser, a survey was performed among nurses, medical technologists, dentists, general practitioners, radiology residents, radiologists, and other medical specialists such as ophthalmologists, surgeons, gynecologists, urologists, pediatricians, and otolaryngologist, among others, at the Clinical Hospital University of Chile. The composition of the survey sample was: 56.7% medical doctors, 26.9% medical technologists, 7.4% nurses, 3% dentists, 3% medical informatics, 1.5% biochemists, and 1.5% students. On the other hand, the sample was distributed as 31.4% male, 34.3% female, and 34.3% unknown gender.

The invitation to participate was shared through digital media (electronic mailing and social networks). All participants were invited to, in turn, share the link to the survey with healthcare professionals of their working and social network. The survey was applied between November and December 2020. In total, 67 subjects completed the survey. For the questions “in which cases would a CBIR system be useful?” and “in which cases would an intelligent interactive visual browser be useful?”, respondents were asked to choose from six possible answers: medical education; research; clinical care; innovation/technological development; personal study; management statistics. 

**Figure 4:**
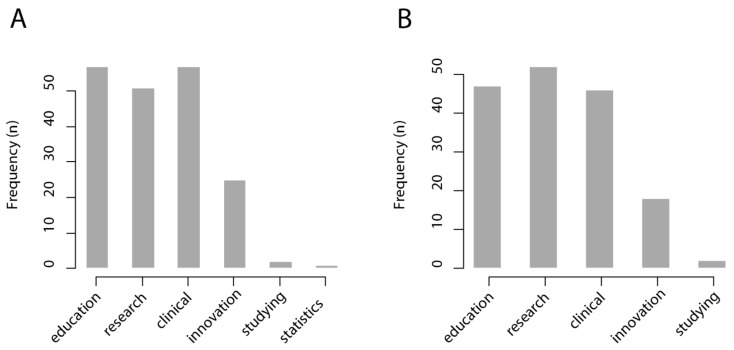
Results of the survey among professionals of the healthcare sector (*n* = 67). Medical education, research, and clinical care were considered the most relevant use cases for (**A**) a content-based medical image retrieval system and (**B**) an intelligent interactive visual browser.

We found that most respondents from the healthcare sector foresee that a CBIR system would be useful for medical education (57/67 = 85%), research (51/67 = 76%), and clinical care (57/67 = 85%), while it would be less useful for innovation and technological development (25/67 = 37%), personal study (2/67 = 0.3%), and management statistics (1/67 = 0.1%). Similarly, most respondents from the healthcare sector foresee that an intelligent interactive visual browser would be useful for medical education (47/67 = 70%), research (52/67 = 78%), and clinical care (47/67 = 70%), while being less useful for innovation and technological development (18/67 = 27%) and personal study (2/67 = 0.3%). The results of this survey are shown in Figure 4.

To further evaluate the system’s usability, the System Usability Scale will be applied in upcoming evaluations of the system once it is implemented and running in a corresponding environment for medical education, research, and care purposes.

## 3. Discussion

### 3.1. State-of-the-Art Image Content-Based Search Engines in the Medical Field 

An ever-increasing amount and quality of digital medical imaging has driven the call and international efforts for the building of large-scale medical image repositories that may unleash unprecedented possibilities for medical education, research, and care purposes. In turn, the burst of medical image analytics capacity due to advances in computer science, computer vision, and deep learning has redeemed novel technological capabilities for developing advanced CBIR systems over the past few decades. These systems are relevant to ease the management of and navigation through large-scale medical image repositories, with long proposed use cases in each of the aforementioned medical purposes [4]. Ahmed et al. [20] proposed a method for merging color and spatial information, achieving excellent results in multiple datasets specialized in texture, color, and morphology. Wei et al. [21] used kernel trick techniques to make the model learn by calculating the Mahalanobis distance, so that it is possible to handle both visual and semantic information. Yet, despite traditional methods having achieved good performance in specific medical contexts, it should be noted that conventional methods for analyzing medical images have reached a point of limited success as they are not capable of tackling the current huge amounts of image data [4]. It is noteworthy that single medical imaging examinations may comprise thousands of images, meaning that massive amounts of images have to be stored in medical image repositories, which challenges the efficiency of CBIR systems for medical purposes and clinical settings [22]. Achieving both accuracy and efficiency to perform real-time image analyses through large-scale repositories such as those currently possible in the era of big data remains a challenge. The problem to tackle nowadays is efficiency, as it is key for ensuring its actual application by end users in medicine. 

Novel techniques leveraging large-scale medical image analysis bring about unprecedented opportunities to minimize the usual trade-off between efficiency and accuracy, paving the pathway towards developing CBIR systems that are both highly accurate and efficient. It has been proposed that reducing the dimensions of feature representation vectors and improving the data indexing strategy may aid on addressing this issue. Yet, it should be noted that good feature representation is essential to accomplish good performance in CBIR systems. Feature representation is categorized into hand-crafted (features are obtained by means of domain expert knowledge) and learned (solely data-driven methods). On the one hand, although hand-crafted features obtained favorable results in CBIR systems, for large-scale medical image settings their major drawback is the need for (expensive) expert knowledge. It is time-consuming, computationally expensive, and the specificity of some hand-crafted methods limits the extension of its use from one medical domain to another [4]. Whereas, on the other hand, for learned features, deep learning does not require domain expert knowledge but only a set of training data to disclose feature representations in a self-learned fashion [23,24]. Many deep learning architectures have been built to map features into abstract representations using manifold neural network layers and numerous parameters [12]. Therefore, having large-scale medical image repositories actually lies in the foundational interests of deep learning-based approaches, as it provides amounts of training data in order of magnitudes that make it possible to optimize the thousands to millions of parameters set up in its neural network’s architectures. Some recent examples of deep learning systems are that of Cai et al. [25], which used a Siamese network to generate a binary embedding through hashing and built a special loss function that allows to distance or approximate such embedding if the pair of input images to the network have the same label or not, generating a robust image recovery system. Next, in 2020, Yang et al. [26], achieved the state-of-the-art method in information retrieval for the public histological dataset *Kimia Path24* [27] using a mixed attention approach that involves both spatial attention and channel attention. 

Supervised and unsupervised deep neural networks have been applied to produce feature representations of medical images. The first demands labeled training data, which, for the case of large-scale medical image datasets, equals expensive physician or domain expert annotation. In order to circumvent these restraints, unsupervised deep neural networks, i.e., auto-encoders, have been proposed for feature representation, for which representation power improves when several auto-encoders are stacked to form deep stacked auto-encoders. Deep belief networks and deep Boltzmann machines are also prevalent deep neural network alternatives for medical image feature representation [10,11], the latter being proposed as a promising solution for next-generation medical image indexing and retrieval systems [4]. It should be realized, however, that learned feature representations are currently used mostly for medical image analytics tasks, but much less frequently for indexation and medical image retrievals tasks [4]. The reason is that, up until relatively recently, most CBIR tasks have been aimed at small-scale image datasets, which inherently prohibits training such deep learning neural networks, whereas domain expert-designed features reach good performance in datasets of this order of magnitude. Nowadays, besides the aforementioned limitations in cases of large-scale datasets of medical images, deep learning-based methods allow the learning of different types of features compared to domain expert-designed features, further underscoring the potential of using deep learning-based methods for the design of CBIR systems in the medical field. 

The deep learning neural network used was CE-Net [13], which is an encoder-decoder architecture. The Xception [14] architecture was used in tandem. As this incorporates convolutional layers that allow the capturing of long-range dependencies, this architecture obtains good results in classification problems. Yet, since this architecture has many parameters, it requires data volumes in order of magnitudes unlikely to be reached in the medical field in the near future. Hence, a different approach was introduced to reduce this gap, i.e., the latent representations created by the CE-Net architecture (images with reduced variability) were used as pre-trained vectors to adjust the Xception architecture (with improved generalization ability) according to diagnosis. 

As a result, with our state-of the-art approach, while CE-Net allows different types of medical images to be represented in the same representation space, the Xception network helps to separate them by diagnosis. It is possible to compute and rank the distance between the query image and the feature vectors (images) in the database, to display the search results on a near neighbor or similarity map, which we have called a proximity graph.

### 3.2. Applications in Medical Education, Research and Care

The previous literature has highlighted education as the major potential beneficiary of image CBIR systems. Such systems would allow medical students, interns, or specialists-in-training (e.g., radiology residents) to query a particular case encountered during their studying, practicing, or training schemes by using the system to retrieve similar cases and related metadata for consideration and instruction. In particular, the intelligent interactive visual browser developed in this project allows apprentices to browse through available images in an easy, intuitive, and straightforward fashion, wherein similar cases are organized and showcased according to their similarity. This possibility is expected to support and enhance self-studying potentiality, and it may also help the down-turning negative effects of otherwise impaired mentor–mentee interactions due to unforeseen circumstances, such as that of the current COVID-19 pandemic. We expect usability to be highly increased, particularly in training and practicing scenarios, by integrating our system into clinical workstations, allowing the direct exportation from the PACS without the need for saving or transforming the original clinical image. Taking into account the importance of easing integration to the PACS and electronic healthcare records, we have developed a fully interoperable repository, which adheres to international standards and integral terminologies. Next, medical lecturers may likewise see potential educational applications. Instructors can easily find medical cases for teaching while graphically displaying potential differential diagnoses of a particular case. This would allow comparing of different annotations for otherwise visually similar medical cases. These are all use cases not previously possible by means of conventional text-based search engines and display. It should also be underscored that in integrated scenarios, trainees would be able to tailor the study of clinical differential diagnoses to those epidemiologically relevant as available in the local archives. This is in line with the contemporary understanding that the unfolding age of artificial intelligence-augmented radiology may enable precision medicine but also precision medical education, fitting trainee-oriented instruction to individual needs [28,29]. Of note is that case-based analyses of relevant differential diagnoses, also referred to as the bottom-up strategy, has been shown to be more effective in comparison to its top-down counterpart (i.e., first, learning diagnostic processes and then practicing), it is in line with adult learning theories, and, importantly, it is also the preferred method used by students and trainees [30]. In summary, case-based radiology learning using our CBIR systems integrated to the PACS may aid medical educators to overcome the burden of preparing laborious training cases while immediately linking metadata generated in real clinical scenarios, and provide specific healthcare facility-relevant experience of commonly encountered similar cases. 

Similarly, for research purposes, it is noteworthy that medical image scientists would usually browse large-scale medical imaging repositories at different—not necessarily diagnostic—levels. Text-based limited search engines cause higher browsing-related time as they force the researcher to implement empirical repository exploration approaches, repeatedly revising and improving query commands until obtaining content-relevant images. Moreover, complementary visual image content- and text-based searches may supply research with the ability to navigate similar cases within a keyword-prioritized or keyword-restricted search context. This searching strategy may allow unprecedented content-based similarity analyses within the boundaries of a particular diagnosis, aiding on, for instance, the study of visual features characterizing different stages or variations of a particular disease [5]. In clinically integrated scenarios, researchers may also take advantage of the implicit (non-textual) information stored in images, mining together image content with its accompanying metadata, just as routinely produced in the clinical context. In the era of big data, such novel research settings may advance medical knowledge by empowering the ability to link visual features with radiological reports, electronic healthcare records, and patients’ outcomes. For otherwise similar cases (i.e., imaging cases with equivalent radiology descriptions and diagnosis), novel research opportunities may be introduced to identify imaging biomarkers that inversely associate with adverse clinical events. This patient-centered approach is in line with the current efforts of providing patient-centered, personalized medicine for value-based healthcare [31,32]. 

For real-time clinical decision support and diagnostic purposes, it is well known the problem-solving potential of having a repository with medical image cases for consultation. Yet, such potential is generally frustrated by the laborious and time-consuming task of navigating textbooks or imaging repositories for that purpose in a fast-paced clinical environment. Such time-constrained setting, e.g., that of current radiology practices, would benefit by having the means to access automatic, independent medical inter-consultations according to medical image content with similar cases available in local archives. For clinical decision support, it has been proposed that typical computer-aided diagnosis systems usually value the probability of disease types, which may not be suitable for imaging specialists whose training was based on reading many images, whereas providing reference images that are perceptually similar could supplement numerical outputs and better fit imaging specialists’ clinical image reading processes. Indeed, it has been underscored that a CBIR system may further aid diagnosis, particularly in cases for which the diagnosis greatly depends on visual features, aligning with evidence-based radiology [33,34,35] and case-based reasoning [30,36] frameworks. In agreement with this, some CBIR systems have recently received FDA clearance for machine learning-driven diagnosis [37,38]. SYNAPSE automatically segments potential lung cancer lesions and retrieves similar cases, whereas another system [39] retrieves nodular and diffuse opacities in lung CT imaging examinations. Recent studies have investigated the application perspectives into radiology, reporting that by integrating pixel-based and metadata-based image feature analyses, CBIR systems may lead to significant practical advances impacting permeation and integration of these methods into clinical and imaging workflow [40]. 

### 3.3. Integration into PACS and Electronic Patient Records, and Anonymizer Service

Particularly in radiology, it has been highlighted that integration with DICOM-based PACS networks is needed to provide functionality in a seamless manner and reach a wider audience of users [41]. It should be realized that a PACS is a rich data source as it stores radiological data generated on a daily basis for clinical purposes, yet this gigantic collection of past radiological cases is rarely used as a searchable repository to enrich ongoing diagnostic capability or solve future diagnostic matters. It is well known that, since the early days of the PACS, radiologists highly regarded the introduction of this system into their workflow as it allowed for image manipulation but also eased retrieval and comparison [42]. Our system may take the retrieval and comparison potential of the PACS one step further into the direction radiologists value for clinical purposes. Because integration is such an important feature, we developed a highly interoperable system to ease integration with any system adhered to international interoperable standards. Furthermore, we designed the repository of the CBIR system with an automatic anonymizer service in order to adhere with international standards on patients’ data privacy policy and to ease multicenter collaboration. The need for multicenter or international collaboration may be foreseen considering that the performance of the CBIR system improves with the size of its imaging repository. 

### 3.4. Acceptance of This Technology in the Clinical Domain 

One important matter in image retrieval research is the evaluation of the behavior of the retrieval system by end users. This is not an easy task, taking into account that one of the most relevant factors for acceptance of this technology in the clinical domain is the perceptual similarity of the retrieved images, which is judged by imaging specialists partially on the basis of subjective factors and may showcase low interobserver agreement; however, it has been recently shown that perceptual or subjective similarity is a solid concept, which can be reliably measured and evaluated [37]. Hence, further research is needed to study the hypothesis that the system that we developed performs adequately for acceptance in the clinical domain. Furthermore, with an extension of the imaging repository to different set of clinical images, it is also warranted to evaluate whether particular image search engines may need to be developed and used for particular anatomical regions or imaging modalities [43].

### 3.5. Limitations of the Proposed System

One of the main limitations of our system is its dependency on two types of grounded datasets. Although the proposed approach is unsupervised in the ranking phase, the representational framework requires tagged data for the training phase. Accordingly, it can be considered a hybrid approach, with a supervised representation learning phase and a ranking phase based on an unsupervised query engine. Both Ce-Net and Xception require grounded images. In the case of Ce-Net, medical images with their respective masks are required. These masks could be expensive to obtain and could eventually limit the ability to incorporate new medical exams into the system. The Xception requires diagnosis labels. In addition, a tiny volume of tagged data involves the risk of overfitting. To avoid these drawbacks, data augmentation and semi-supervised learning techniques are recommended since they would facilitate the training of the representation learning framework.

## 4. Conclusions

We developed a deep learning-based CBIR system and a first-of-its-kind intelligent visual browser that interactively displays on a similarity map a set of imaging examinations with similar visual content, making it possible to search for and efficiently navigate through a large-scale medical imaging repository, even if it has been set with incomplete and curated metadata. The system was fashioned with an anonymizer service and designed to be fully interoperable according to international standards in order to stimulate its integration within healthcare systems and its adoption for medical education, research, and care. Professionals of the healthcare sector, by means of a self-administered questionnaire, underscored that this CBIR system and intelligent interactive visual browser would be highly useful for these purposes. Further studies are warranted to complete a comprehensive assessment of the performance of the system through case description and protocolized evaluations by medical imaging specialists.
